# Successful heart transplantation after prolonged cardiac arrest and extracorporeal life support in organ donor–a case report

**DOI:** 10.1186/s13019-015-0393-8

**Published:** 2015-12-18

**Authors:** Diego Arroyo, Yvan Gasche, Carlo Banfi, Brian Stiasny, Karim Bendjelid, Raphaël Giraud

**Affiliations:** Intensive Care Unit, Geneva University Hospitals, Rue Gabrielle Perret-Gentil 4, CH-1211 Geneva, Switzerland; Division of Cardiovascular Surgery, Geneva University Hospitals, Geneva, Switzerland; Faculty of Medicine, University of Geneva, Geneva, Switzerland; Geneva Hemodynamic Research Group, Geneva, Switzerland; Divisions of Pediatric Cardiology and Children’s Research Centre, University Children’s Hospital Zurich, Geneva, Switzerland

**Keywords:** E-CPR, ECLS, ECMO, Heart transplantation, Refractory cardiac arrest

## Abstract

**Background:**

Although heart transplantation is a successful therapy for patients suffering from end-stage heart failure, the therapeutic is limited by the lack of organs. Donor cardiac arrest is a classic hindrance to heart retrieval as it raises issues on post-transplant outcomes.

**Case presentation:**

The present case reports a successful heart transplantation after prolonged donor cardiac arrest (total lowflow time of 95 minutes) due to anaphylactic shock necessitating extracorporeal life support. We further provide an overview of the current evidence and outcomes of heart transplantation in cases of donor cardiac arrest.

**Conclusion:**

Providing that donor and recipient criteria are respected, donor cardiac arrest does not seem to be an adverse predictor in heart transplantation.

**Electronic supplementary material:**

The online version of this article (doi:10.1186/s13019-015-0393-8) contains supplementary material, which is available to authorized users.

## Background

Heart transplantation is an effective therapy for patients suffering from end-stage heart failure, but the procedure is limited by the shortfall in the availability of organs. Although demand is likely to continue to increase, the number of heart transplantations has decreased in the past decades [1] pushing the limits in exploring, evaluating and resuscitating “borderline” donor organs. In specific settings, even hearts with suboptimal function may be transplanted with similar mortality rates than those with normal function [2].

## Case presentation

A 17-year-old female with a history of allergy and systemic reactions to peanuts unknowingly ate a plate of Spaghetti in a Chinese restaurant cooked with groundnut oil. She immediately developed an anaphylactic shock with dyspnea, agitation and cardiac arrest. Cardiopulmonary resuscitation (CPR) was begun upon arrival of the medical emergency team, 10 minutes after collapse-the first rhythm was asystole. Intravenous Epinephrine (total dose 10 mg) and fluid administration (total 2.5 L) were administered and although transient return to spontaneous circulation was achieved at 22 minutes, the patient rearrested with recurrent pulseless electrical activity and then asystole. Only mild laryngeal edema was noted on intubation. She was transferred to our tertiary care facility, under continuous CPR with the Lund University Cardiac Arrest System–Version 2 device (LUCAS 2; Jolife AB, Lund, Sweden). Immediate femoro-femoral, veno-arterial extra-corporeal life support (ECLS) was surgically implanted (RotaFlow, Maquet, Hirrlingen, Germany). No-flow was estimated to be 10 minutes and total low-flow from CPR initiation to full ECLS support 95 minutes. Soon after the establishment of the VA-ECLS, the patient regained an effective sinus rhythm. However, the transesophageal echocardiography performed during the implantation procedure showed a bilateral ventricular dysfunction with global hypokinesia (Additional file 1). In order to maintain both a left ventricular drainage and a mean arterial pressure (MAP) above 65 mmHg, a hemodynamic support with dobutamine and norepinephrine was necessary during the first 12 hours. There was no sedative or analgesic administration at any point during pre- or in-hospital care.

Clinically, the patient was deeply comatose-Glasgow Coma Scale 3–with bilaterally dilated pupils unresponsive to light. Complete clinical examination and brain death assessment were performed according to the guidelines of the Swiss Academy of Medical Sciences [3]. The apnea test was carried out in accordance with previously published data on apnea test and brain death testing in patients under ECLS [4]. The clinical diagnosis of brain death was established 16 h after hospital admission and the parents consented to organ donation. Thyroid hormones, low dose corticosteroids and desmopressin were administered for the organ preservation and diabetes insipidus.

Cardiac assessment for heart donation was made by transthoracic echocardiography (TTE). Given the young age, coronary angiography was not performed. TTE displayed normal findings with no inotropic or vasoactive support and ECMO flow lowered to 1.5 L / min (Additional file 2). Left ventricular function was assessed by measuring the left ventricular outflow tract velocity time-integral (16 cm), visual assessment of the left ventricular ejection fraction (60 %) and measurement of the systolic peak velocity of the mitral annulus (TDSa) (> 6 cm/s). Right ventricular function was assessed visually and by tricuspid annular plane systolic excursion (TAPSE) (18 mm). There were no segmental anomalies as well as normal valves. The heart was retrieved 50 h after hospital admission (total ECLS support of 50 h, ECLS support prior to organ assessment 16 h and time from assessment to transplant 34 h). Warm and cold ischemia times were 23 and 202 minutes respectively. The heart was transplanted in an 11-year-old female with anthracyclin-induced cardiomyopathy following treatment for high-grade osteosarcoma of the tibia. The immediate post-operative course was favourable allowing extubation on day 2, she required milrinone but no mechanical support for a total intensive care length of stay of 21 days. The patient was discharged 56 days post transplantation, she is symptom-free at 6-month follow-up with normal cardiac function.

## Discussion

Medical teams may be reluctant to heart retrieval from resuscitated donor cardiac arrest due to concerns on post-transplant outcomes. The critical question is if these hearts can resist the ischemic injury of procurement, storage and transportation.

The impact of donor cardiac arrest on heart transplantation was studied in a retrospective cohort analysis of 19′980 cardiac transplantations form the United Network for Organ Sharing Registry [5]. The 856 cases with donor history of cardiac arrest (median duration of arrest time 15 min) had unadjusted 30-day, 1-, 5-and 10-year actuarial survival rates similar to the non-arrest group. Patients receiving hearts with short arrest duration (0–8 min) had significantly improved survival rates as compared with all other patients including those from the non-arrest group. It was hypothesized that ischemic preconditioning may explain this observation. However, an increase in the duration of arrest time (> 25 min) was associated with decreased survival. Cardiac arrest did not negatively influence survival after heart transplantation in a cohort of 604 patients, 38 of which were transplanted with hearts from donors who were resuscitated after a cardiac arrest of mean duration of 15 min [6]. The one-and 5-year survival comparing arrest and non-arrest groups was 94.2 % vs. 83.6 % and 79.8 % vs. 74.5 % respectively (*p =* 0.35). Donor cardiac arrest was not an adverse predictor of mortality on multivariate analysis.

Furthermore, a large multicentre transplant database of the 29,242 adult heart transplantations in which 1,396 patients (4.7 %) received hearts from CPR donors did not show inferior outcomes at 5-year follow-up in recipients of heart transplantation from selected CPR donors [7].

However, none of the patients in the above described cohorts benefited from ECLS. The issue of extracorporeal support and heart transplantation has been addressed in a case series from Taiwan in which 5 donors after brain death were put on ECLS due to acute hemodynamic instability. Of these, 3 hearts were harvested with uneventful outcomes in the recipients [8].

The management of endocrine dysfunction in potential heart donation is of particular interest. The deterioration of cardiac function and need for inotropic support could be reversed by T3 supplementation. [9] A retrospective analysis based on 63,593 donors showed that T3/T4 therapy results in more transplantable organs, with no decrease in post transplantation graft survival [10]. Corticosteroid administration is associated with significant reductions in early graft dysfunction in heart transplantation [11] and desmopressin can improve the number and quality of retrieved organs. [12]

Transthoracic echocardiography is the preferred imaging method for donor heart assessment [13]. It is paramount that patients under ECLS meet the qualitative and quantitative echocardiographic weaning criteria [14]. We believe that the positive outcome was not only due to the respect of standard donor and recipient criteria but also because of a careful selection process combining clinical and echocardiographic assessment. The complete recovery of the myocardium may have been due to the reversible nature of the cause. Anaphylactic shock-associated cardiomyopathy results in acute heart failure secondary to severe coronary vasospasm and usually recovers quickly without major sequelae [15, 16]. The initiation of ECLS provided timely hemodynamic support and may well have speeded coronary perfusion optimisation and left ventricular function recovery. The satisfactory TTE findings and the young age of the patient, prompted consideration for organ donation.

To date, there is minimal data on ECLS in donor cardiac arrest and heart transplantation. The use of ECLS in the present case was for the sole attempt of saving the patient and the unfortunate cerebral insult that ensued shifted the goals towards optimisation of potentially transplantable organs. The question of ECLS in heart donation after cardiac arrest is complex and raises both technical (timely cannulation with exclusion of the cerebral circulation) and major ethical issues (the donor ceases to be “non-heart beating” once the coronary circulation is restored). Whether or not this is a potential field for donor heart expansion is uncertain. The cost effectiveness is of course debatable and will vary between health systems.

Finally, the cause of cardiac arrest-the peanut allergy-should not be overlooked as the allergy may be “transmitted” to the recipient with major consequences [17].

## Conclusion

Providing donor and recipient criteria are carefully respected, heart transplantation may be successful even after prolonged donor cardiac arrest.

## Additional files

Additional file 1:
**Transesophageal echocardiography (transgastric short axis view 0°) during VA-ECLS implantation immediately after restoration of sinus rhythm showing severe left ventricular systolic dysfunction.** (AVI 9299 kb) Additional file 2:
**Transthoracic echocardiography (parasternal short axis view) after brain death confirmation showing normal left ventricular systolic function (VA-ECLS with flow at 1.5 L/min).** (AVI 9246 kb)
